# Biological Markers of Myeloproliferative Neoplasms in Children, Adolescents and Young Adults

**DOI:** 10.3390/cancers16234114

**Published:** 2024-12-08

**Authors:** Aleksandra Ozygała, Joanna Rokosz-Mierzwa, Paulina Widz, Paulina Skowera, Mateusz Wiliński, Borys Styka, Monika Lejman

**Affiliations:** 1 Independent Laboratory of Genetic Diagnostics, Medical University of Lublin, 20-093 Lublin, Poland; aleksandra.ozygala@uszd.lublin.pl (A.O.); paulina.skowera@umlub.pl (P.S.); borys.styka@umlub.pl (B.S.); 2 Department of Genetic Diagnostics, University Children’s Hospital, 20-093 Lublin, Poland; joanna.rokosz-mierzwa@uszd.lublin.pl (J.R.-M.); mateusz.wilinski@uszd.lublin.pl (M.W.); 3 Student Scientific Society of Independent Laboratory of Genetic Diagnostics, Medical University of Lublin, 20-059 Lublin, Poland; paulina.widz@wp.pl

**Keywords:** myeloproliferative neoplasm, children, chronic myeloid leukemia (CML), polycythemia vera (PV), essential thrombocythemia (ET), primary myelofibrosis (PMF), chronic neutrophilic leukemia (CNL), chronic eosinophilic leukemia (CEL), juvenile myelomonocytic leukemia (JMML)

## Abstract

Myeloproliferative neoplasms (MPNs) are clonal hematopoietic cancers characterized by hyperproliferation of the myeloid lineages. These clonal marrow disorders are extremely rare in pediatric patients. MPN is reported to occur 100 times more frequently in adults, and thus research is primarily focused on this patient group. At present, modern diagnostic techniques, primarily genetic, facilitate the identification of the biology of these diseases. The key genes are *JAK2*, *MPL*, and *CALR*, namely, driver mutations, which are present in approximately 90% of patients with suspected MPN. Moreover, there are more than 20 other mutations that affect the development of these hematological malignancies, as evidenced by a review of the literature. The pathogenic mechanism of MPNs is characterized by the dysregulation of the JAK/STAT signaling pathway (*JAK2*, *MPL*, *CALR*), DNA methylation (*TET2*, *DNMT3A*, *IDH1/2*), chromatin structure (*ASXL1*, *EZH2*), and splicing (*SF3B1*, *U2AF2*, *SRSF2*). Although rare, myeloproliferative neoplasms can involve young patients and pose unique challenges for clinicians in diagnosis and therapy. The paper aims to review the biological markers of MPNs in pediatric populations—a particular group of patients that has been poorly studied due to the low frequency of MPN diagnosis.

## Simple Summary

Myeloproliferative neoplasms (MPNs) are exceedingly rare in children, adolescents, and young adults. The current literature focuses mainly on older patients, so the purpose of the review is to present the genetic landscape of pediatric patients. Modern genetic diagnostics makes it possible to identify genetic markers involved in the pathomechanisms of these uncommon clonal marrow disorders. Excluding driver gene mutations (*JAK2*, *MPL*, *CALR*), over 20 other mutations have been described as of significance to MPNs. The aim of the review is to improve and present knowledge of MPNs and genetic targets for diagnosis and therapy in children, adolescents, and young adults.

## 1. Introduction

Myeloproliferative neoplasms (MPN) are predominantly diagnosed in adults around the age of 60. The incidence of MPN in pediatric patients is approximately 0.82 per 100,000 individuals per year, while in adults, MPNs are reported to be 100 times more prevalent. A hallmark symptom of MPN is splenomegaly; however, it is noteworthy that not all pediatric patients present with clinical manifestations. Symptoms such as abdominal discomfort, bone pain, persistent fatigue, difficulty concentrating, and pruritus may be observed, but a significant proportion of patients remain asymptomatic [1]. Children do not represent a large cohort of patients with MPNs. A better understanding of MPNs’ etiology in children and their biological markers will increase diagnostic knowledge and vigilance among clinicians. A distinctive characteristic of myeloproliferative neoplasms, with the notable exception of chronic myeloid leukemia, is the absence of the Philadelphia chromosome. MPNs are characterized by the presence of somatic mutations in key genes, including *JAK2*, *MPL*, and *CALR*, which account for approximately 90% of cases. Additional markers associated with these tumors include genes involved in epigenetic modification, such as *ASXL1*, *EZH2*, *IDH1*, *IDH2*, *TET2*, and *DNMT3A*, as well as genes implicated in mRNA splicing, namely, *SF3B1* and *U2AF2*. The identification of these mutations has not only advanced our understanding of MPN biology but has also facilitated the development of targeted therapeutic approaches [2,3,4].

## 2. Classification of MPN

In the year 2022, the two most prevalent classifications of myeloid neoplasms underwent a process of revision: the 5th World Health Organization (WHO) classification system and the International Consensus Classification (ICC). The primary classifications of myeloproliferative neoplasms (MPN) have remained largely consistent with previous editions. The initial diagnostic assessment of MPN is primarily reliant on a detailed integration of clinical characteristics, molecular diagnostic results, and typically a morphological examination of a trephine bone marrow biopsy. Most patients with MPN are diagnosed during the chronic phase (CP), which has the potential to progress to a blast phase (BP) characterized by the accumulation of secondary cytogenetic and/or molecular abnormalities. The present study will focus exclusively on the category of myeloid neoplasms (without MDS, MDS/MPN), as illustrated in Table 1 and Table 2 [5,6].

Minor revisions have been made to the diagnostic criteria for *BCR*::*ABL1*-negative myeloproliferative neoplasms. The classification continues to focus on the differentiation between polycythemia vera (PV), essential thrombocythemia (ET), and primary myelofibrosis (PMF) by utilizing diagnostic criteria established in earlier editions, with some minor adjustments. The differentiation among these neoplasms is based on the comprehensive integration of findings from peripheral blood analyses, molecular data, and morphological evaluations of bone marrow. This approach is essential, as no single parameter offers adequate diagnostic specificity on its own.

## 3. Biological Markers

Genetic mutations play an important role in the pathogenesis of MPN. In pediatric patients, the specific genetic alterations associated with MPN remain less clearly defined compared to those in adult patients. For children suspected of having MPN, comprehensive bone marrow assessments are conducted alongside the evaluation of hematological parameters. Genetic testing is a critical component of the diagnostic process; its primary objective is to identify mutations that not only serve as driver mutations but also influence therapeutic strategies by providing potential targets for treatment. In their review, Kucine et al. proposed a diagnostic panel comprising various tests for children suspected of MPN. This panel includes obtaining a thorough medical history and conducting a physical examination, in addition to laboratory tests such as complete blood count, erythropoietin levels, lactate dehydrogenase (LDH), iron studies, ferritin, total iron binding capacity (TIBC), hepatic panel, and von Willebrand panel, particularly in patients exhibiting thrombocytosis (>1000 × 10^9^/L) or bleeding symptoms. Bone marrow investigations encompass immunophenotyping of marrow cells, biopsy for assessment of iron stores, morphological examination, fibrosis evaluation, and cellularity analysis. Genetic studies focus on the three most relevant genes implicated in the pathomechanism of MPN: the driver mutations *JAK2*, *MPL*, and *CALR*. The pathogenic mechanism is characterized by the dysregulation of the JAK/STAT signaling pathway [3]. Additionally, genes implicated in DNA methylation, including *TET2*, *DNMT3A*, *IDH1*, and *IDH2*, as well as those linked to maintenance of chromatin structure such as *ASXL1* and *EZH2*, and splicing factors *SF3B1*, *SRSF2*, and *U2AF1*, have also demonstrated mutations in MPN [2]. Further research is warranted to elucidate the genetic predispositions that may facilitate the transformation of leukemic MPNs into acute myeloid leukemia (AML), with emphasis on genes such as *ASXL1*, *IDH1*, *IDH2*, and *SRSF2*, among others [7]. The somatic mutations are characterized in Table 3 and signaling MPN driver mutations are additionally described below.

### 3.1. JAK2

The *JAK2* gene is situated on the short arm of chromosome 9 (9p24). It encodes an intracellular protein that is involved in the signaling pathways of erythropoietin and thrombopoietin. *JAK2* is a member of the Janus kinase family and comprises two distinct domains: the JH1 domain at the C-terminus, which exhibits tyrosine kinase activity, and the JH2 domain, often referred to as the pseudokinase domain. The mechanism by which *JAK2* influences hematopoietic cells operates as follows: bone marrow cytokine receptors, upon receiving stimuli, activate Janus family kinase (JAK) proteins. These proteins subsequently phosphorylate various cytoplasmic proteins, including signal transducers and activators of transcription (STAT), thus forming one of the principal JAK-STAT signaling pathways. In 2005, a somatic mutation in the *JAK2* gene was identified in patients with myeloproliferative neoplasms (MPN). This mutation was found in a significant proportion of patients, prompting extensive research interest. The mutation is a non-synonymous point alteration involving a guanine to thymine substitution in exon 14 of the *JAK2* gene (G1849T, c.1849G > T). This results in an amino acid substitution where valine is replaced by phenylalanine at position 617 (V617F, p.Val617Phe), located in the JH2 domain. The presence of this mutation in the pseudokinase domain effectively disrupts the inhibitory effect that this domain normally exerts on the catalytic activity of the kinase, leading to constitutive activation of the *JAK2* tyrosine kinase. Consequently, there is an independent and excessive proliferation of hematopoietic cells in the bone marrow, as the JH1 domain is no longer inhibited by the JH2 pseudokinase. This aberrant activation promotes the phosphorylation and subsequent activation of STAT transcription factors [8,9,43,44,45,46,47]. Alterations in exon 12 of the *JAK2* gene occur less frequently and typically involve the substitution of leucine for lysine at position 539. Compared to the V617F mutation, changes within exon 12 are associated with enhanced *JAK2* signaling [10]. The prevalence of *JAK2* mutations in children with MPN is significantly lower than that observed in older patients, reinforcing the notion that somatic mutations are acquired with advancing age. Additionally, *JAK2* V617F mutations of prenatal origin have also been detected [48].

### 3.2. MPL

A year after the identification of the *JAK2* mutation as a pivotal factor in the pathogenesis of myeloproliferative neoplasms (MPN), researchers began to investigate patients lacking *JAK2* alterations. This inquiry led to the discovery of another significant mutation located within exon 10 of the *MPL* gene, which resides on chromosome 1 at locus 1p34.2. The *MPL* gene encodes the thrombopoietin (TPO) receptor. These alterations primarily manifest as missense point mutations at codon 515 (W515L, W515K) and, to a lesser extent, at codon 505 (S505N) of the *MPL* gene [2,12,13]. These mutations result in persistent ligand-independent activation of the receptor, which is crucial for the growth and proliferation of platelets and megakaryocytes. The expression of *MPL* regulates TPO levels; mature platelets facilitate a negative feedback mechanism by sequestering TPO that binds to the *MPL* receptor (TPO-R). Mutations in *MPL* lead to an altered expression profile in megakaryocytes and platelets, resulting in an increased proportion of TPO remaining bound to its receptors. This, in turn, promotes enhanced proliferation of megakaryocytes. The presence of *MPL* mutations in platelets disrupts the effective regulatory mechanism for TPO homeostasis, ultimately resulting in thrombocytosis. The identification of mutations at codons 515 or 505 of the *MPL* gene, particularly in the absence of the *JAK2* p.V617F mutation, holds clinical significance for the diagnosis of essential thrombocythemia (ET) or primary myelofibrosis (PMF), especially in patients presenting with relevant symptoms and clinical features [43,44,45,46]. In cases where *MPL* mutations are homozygous, there is an increased susceptibility to the development of bone marrow fibrosis. *MPL* alterations contribute to excessive proliferation of the myeloid lineage, as signaling through the JAK-STAT pathway is augmented by the sustained activation of the JAK2 receptor [2,9].

### 3.3. CALR

The *CALR* gene, located at locus 19p13.13, encodes the protein calreticulin, which serves as an endoplasmic reticulum chaperone. This cytoplasmic protein is characterized by a specific C-terminal amino acid sequence known as KDEL. The C-terminal domain of calreticulin functions as a primary calcium-binding and storage protein within the tubules of the endoplasmic reticulum and the nucleus of the cell [2,9,15,16,17,18,43,44,45].

In 2013, two independent studies identified mutations in the *CALR* gene among patients with primary myelofibrosis and spontaneous hyperplasia. In approximately 85% of these patients, a 52-base pair deletion was observed in exon 9 (c.1099_1150del), leading to a type I variant, p.Lys367Thrfs46. Alternatively, a TTGTC insertion in the same exon (c.1154_1155ins) resulted in a type II variant, p.Lys385Asnfs47. Both mutations induce a frameshift by one base pair, resulting in premature termination of protein translation. Notably, the resulting transcript is resistant to degradation via the nonsense-mediated decay (NMD) pathway, leading to the loss of part (in type II mutations) or the entirety (in type I mutations) of the positively charged amino acid sequence located at the C-terminus of calreticulin (KDEL).

The absence of the carboxyl terminus results in the elimination of the retention signal necessary for calreticulin’s localization within the endoplasmic reticulum. The positive charge of this C-terminus is critical for calreticulin’s binding to the thrombopoietin receptor (MPL/TPO-R) and the consequent induction of a proliferative phenotype. The mutations in *CALR* confer a loss of calcium-binding functionality, as the negatively charged C-terminus is replaced by a basic peptide sequence in the mutant protein. Following its binding to the thrombopoietin receptor, mutant *CALR* can be detected at the surface of the cell membrane, presenting itself as a potential therapeutic target. The interaction between *CALR* and *MPL* is vital for the transcriptional activation of the STAT5 transcription factor, which occurs exclusively at the cell membrane. Furthermore, calreticulin binding within the endoplasmic reticulum has been shown to inhibit the phosphorylation and subsequent activation of STAT5 by *JAK2* kinase [2,9,15,16,17,18,43,44,45,46,47].

A total of 36 distinct types of insertions and deletions in *CALR* exon 9 have been documented, each resulting in the generation of a new C-terminal calreticulin peptide. Notably, patients harboring *CALR* mutations have been reported to exhibit higher platelet counts and lower hemoglobin levels compared to those with *JAK2* mutations [2,9,15,16,17,18].

## 4. Chronic Myeloid Leukemia (CML)

Chronic myeloid leukemia (CML) is uniquely characterized among myeloproliferative neoplasms (MPNs) by the presence of a *BCR*::*ABL1* fusion gene, which arises from the translocation of the long arms of chromosomes 9 and 22, specifically t (9;22) (q34;q11). This fusion represents a definitive marker for CML. The *BCR*::*ABL1* fusion protein promotes the proliferation of myeloid lineage cells in the bone marrow due to the constitutive activation of the ABL1 proto-oncogene resulting from this rearrangement. The *BCR*::*ABL1* breakpoints occur within the major breakpoint cluster regions (M-BCR) of the *BCR* gene, specifically located between exons 13 and 15, encompassing an area of approximately 5800 base pairs. This rearrangement leads to the formation of a p210 fusion protein with a molecular weight of 210 kDa. In the *ABL1* gene, breakpoints are identified between exon 1 and exon 2, with DNA breaks in both fusion genes occurring within intronic regions. It is noteworthy that the breakpoints of the fusion gene exhibit differences between pediatric and adult patients; M-BCR is predominantly enriched at the centromere in adults, whereas it is found at the telomeres in children.

The e14a2 transcript of the *BCR*::*ABL1* fusion is more prevalent than the e13a2 transcript; however, e13a2 is observed with a lower frequency in adults (37.9%) compared to children (39.6%). Transcript assembly of the *BCR*::*ABL1* fusion entails the fusion of the *BCR* gene sequence with the second exon of the *ABL1* gene, leading to differences in the length of the resultant *BCR* fusion gene. In pediatric patients, CML is characterized by leukocytosis with a predominance of granulocytic lineage.

CML is considered rare in the pediatric population and accounts for less than 10% of all reported CML, typically presenting between the ages of 11 and 12 years. The CML cases represent 2–3% of childhood leukemias and approximately 9% of leukemias in adolescents aged 15 to 19 years. The annual incidence of CML in patients under 15 years old is reported to be 0.6–1.0 cases per million, while in adolescents, the incidence increases to 2.1 cases per million. The majority of cases occur in males, with estimates ranging from 60% to 77%. The youngest reported patient diagnosed with CML was just 10 months old. Symptoms associated with CML are often nonspecific, including abdominal pain and weakness, leading to frequent incidental diagnosis [35,49,50,51,52,53,54]. CML is classified into three phases: chronic phase (CP), blast phase (BP), and accelerated phase (AP), the latter of which has been omitted from the current WHO classification and is referred to as the “high-risk chronic phase.”

The latest WHO classification introduced the following features in CML-CP associated with increased risk of progression:

At diagnosis:

- High ELTS (EUTOS long-term survival) score;
- 10–19% blasts in the peripheral blood and/or bone marrow;
- ≥20% basophils in the peripheral blood;
- Ph+ cells, including *MECOM* rearrangements, deletion of chromosome 7, isochromosome 17q, complex karyotype;
- Ph+ cells, including additional chromosomes: 8, 19, 21, Ph+, *KMT2A* rearrangements;
- Clusters of small megakaryocytes, associated with significant reticulin and/or collagen fibrosis.

During treatment:

- Resistance to TKI, including loss of prior responses, the emergence of additional cytogenetic abnormalities, and *BCR*::*ABL1* kinase domain mutations.

The ICC in 2022 introduced diagnostic criteria for CML-AP and CML-BP, in which AP is classified for patients with 10–19% blasts in bone marrow or peripheral blood and BP with ≥ 20% [6].

National Comprehensive Cancer Network (NCCN) guidelines recommend the following diagnosis of CML: history and physical examination with determination of spleen size, blood count with cell differentiation, selected blood biochemical parameters, and bone marrow tests. Bone marrow aspirate tests, in addition to morphology (with special attention to blast and basophil percentages), mainly include genetic testing: karyotype, fluorescence in situ hybridization, and RT-PCR to determine *BCR*::*ABL1* profile. Cerebrospinal fluid (CSF) studies are required, but only in suspected CML-BP [55]. Children show higher platelet and white blood cell counts compared to adults with CML. Pediatric patients respond less effectively to imatinib treatment than adults—they are less likely to achieve <10% *BCR*::*ABL1* fusion transcript after 3 months of treatment. Children and young adults are distinguished by more severe chronic phase CML symptoms than adults and a higher frequency of advanced phase at diagnosis. In the blastic phase, children tend to have a more complex karyotype than adults with CML [35,56,57]. In addition to the genetic background of CML concerning the presence of *BCR*::*ABL1*, there are cases of *ASXL1* gene mutation. This mutation is more common in children and young adults than in adults, further highlighting the fact that this marker is studied among the analyzed group of patients. Moreover, it is associated with higher disease risk and worse molecular response in the first stages of treatment. The *ASXL1* gene is located on chromosome 20 (locus 20q11.21) and encodes additional sex combs-like 1 (a protein of 1541 amino acids), the mutation of which leads to loss of control of Polycomb complex 2. This complex controls the methylation of histone H3K27. Mutation of *ASXL1* ultimately leads to the transformation of myeloid cells by inhibiting methylation [35,36,37]. Currently, standard treatment for CML includes tyrosine kinase inhibitors (TKI). The prognosis among children with CML is excellent—a five-year progression-free survival rate of 92%. Response to therapy is determined by hematological, cytogenetic, and molecular results, respectively, normalization of peripheral blood counts, decrease in the Ph-positive chromosomes in bone marrow metaphases, and decrease in the amount of BCR-ABL1 chimeric mRNA using qRT-PCR. So far there are data on weakened immune systems and endocrine disorders along with delayed puberty and impaired growth [35,51,58,59].

## 5. Polycythemia Vera (PV)

Polycythemia vera (PV) is classified as a myeloproliferative neoplasm characterized by the autonomous overproduction of erythrocytes by hematopoietic stem cells (HSC), occurring independently of erythropoietin (EPO) stimulation. Erythrocytosis is caused by the expression *JAK2*, which is correlated with JAK/STAT pathway activation, promoting blood cell proliferation and inhibiting apoptosis. This excessive erythrocytosis can lead to the formation of microclots, which pose significant risks to the patient’s health and may be life-threatening causing thromboembolic, hemorrhagic events.

In pediatric patients, the clinical manifestations of polycythemia vera tend to be nonspecific; the most frequently reported symptoms include headache, dizziness, general weakness, fatigue, and splenomegaly. Characteristic features associated with PV may also include facial and upper extremity erythema, as well as pruritus that intensifies following exposure to warm water. Polycythemia vera is predominantly diagnosed in individuals over the age of 60. Due to its rarity in the pediatric population and the limited number of reported cases, estimating the incidence of polycythemia vera among children presents significant challenges, with pediatric cases accounting for approximately 1% of all PV diagnoses.

Because of the non-specific and initially non-difficult symptoms in children, abnormalities are most often detected by accident during routine blood count tests. The following tests are recommended to make a full diagnosis and characterized in Table 4 [60,61,62,63].

According to the 2022 ICC guidelines [6], the diagnosis of polycythemia vera is conditioned by meeting three major criteria or the first two major criteria and a minor criterion, these are the following:

- Major criteria: increased hemoglobin and/or hematocrit concentration in the blood; absence of *JAK2* V617F or *JAK2* exon 12 mutation; bone marrow biopsy shows hypercellularity with panmyelosis (predominance of erythroid lineage, leukocyte and thrombocyte lineage).
- Minor criteria: low erythropoietin concentration in the blood [5,60].

The 5th WHO edition diagnostic criteria for PV are largely similar to those of the ICC but no longer require RCM assessment [5,60]. Treatment depends on the child’s symptoms, age, and general health. Pediatric patients are mainly in the low-risk group. Due to this, it is advisable to maintain hematocrit below 45% by performing phlebotomy every 2–4 days and to dose aspirin (40–100 mg)/once daily. Venesection is aimed at reducing blood viscosity, thus preventing thrombotic events and their associated complications, while also helping to alleviate symptoms. Allogeneic bone marrow transplantation is one of the treatment methods used in children’s therapy. In patients with confirmed *JAK2* V617F mutation: ruxolitinib, a *JAK1/2* kinase inhibitor, is used in the treatment of polycythemia [61,62,63,64,65,66]. In children, there is a high risk of PV transformation into myelofibrosis or MDS/AML and thrombosis. Patients with PV are also at increased risk of bleeding and stroke. A moderate lifestyle is recommended, as well as avoiding hot baths to prevent unpleasant symptoms of itching or burning of the skin. In the population >65 years of age, survival is similar to that of the general population [60,61,62,63,64].

In the study by Harrison C. et al. conducted on a group of patients aged 18 and older (with a median age of 66), classified as high-risk, the treatment of patients suffering from PV was compared in detail using Ruxolitinib (RUX) versus Best Available Therapy (BAT). The study demonstrated greater treatment efficacy with RUX, as patients in this cohort required fewer venesections also hemoglobin and hematocrit levels were lower. Ruxolitinib was associated with more frequent molecular responses and improved treatment efficacy, but it also led to more frequent infections and hematologic toxicities [66].

The study conducted by Hultcrantz M. et al. on a population of 9429 patients registered in the Swedish database, aged 18 to over 80 years, evaluated the risk of thrombosis in MPN patients. The study showed that the risk of thromboembolic events, including myocardial infarction, ischemic stroke, and pulmonary embolism, was elevated across all age groups, with the highest rates observed in newly diagnosed patients. Key factors influencing the development of thrombosis, in addition to age >60 years in patients with PV, include hematocrit >45%, elevated leukocyte count, concomitant cardiovascular risk factors, and the presence of the *JAK2* V617F mutation. Patients with the *CALR* mutation demonstrated a lower risk of thrombosis.

Treatment with *JAK1* inhibitors, primarily, ruxolitinib and interferon, may be effective in further reducing the risk of thrombosis. During the 5-year follow-up, the risk of both arterial and venous thrombosis gradually decreased, but it remained higher compared to the control group. The study also emphasized that while the risk of venous events was greater, arterial events occurred twice as often in MPN patients [67].

## 6. Primary Myelofibrosis (PMF)

Primary myelofibrosis (PMF) is one of the rarest myeloproliferative neoplasms, defined by marrow fibrosis with secondary formation of extramedullary foci of hematopoiesis. The foci of metaplasia are primarily located in the spleen and liver, resulting in progressive enlargement of these organs (spleno- and hepatomegaly). The incidence rate of PMF has been observed to vary between 0.8 and 2.1 cases per 100,000 individuals per year, with no discernible gender-based difference. The median age at diagnosis is approximately 65 years, with a small percentage of patients (approximately 10%) exhibiting features of PMF development prior to the age of 45 [68,69,70,71,72].

Primary myelofibrosis presents with a range of clinical manifestations and courses. The previous edition of the WHO classification, published in 2016, distinguished the prefibrotic form of PMF (pre-PMF) as a discrete disease entity. The diagnosis of pre-PMF and PMF is based on the fulfillment of strict diagnostic criteria, which are divided into major and minor criteria. The greater criteria for the prefibrotic phase of myelofibrosis (pre-PMF) are as follows:

- Proliferation and atypia of megakaryocytes (small to large forms, with an abnormal nucleus-to-cytoplasm ratio and hyperchromatic, atypical nuclei). Megakaryocytes form dense clusters without reticulin fibrosis > grade 1, with increased marrow cellularity, granulocytic lineage proliferation, with frequent suppression of erythropoiesis.
- No WHO criteria for diagnosis: PV, ET, CML, MDS or other myeloid malignancies.
- Presence of mutations in one of the genes: *JAK2*, *CALR*, *MPL*, or other clonal growth markers (e.g., *MPL* p.W515L/K). In the absence of a clonality marker-exclusion of fibrosis in the bone marrow due to inflammatory or other malignant disease.

Minor pre-PMF criteria include the following:

- Anemia unrelated to comorbidities.
- Leukocytosis (≥11 × 10^9^/L).
- Palpable spleen.
- Increase in lactate dehydrogenase (LDH) activity.

Conversely, the following major criteria are employed to diagnose PMF in the marrow fibrosis stage:

- Megakaryocyte proliferation and atypia are associated with grade 2 or 3 reticulin and/or collagen fibrosis.
- No WHO criteria for diagnosis: CML, PV, ET, MDS, or other myeloid malignancies.
- The presence of the *JAK2* p.Val617Phe, *CALR*, or *MPL* mutation, or the absence of these mutations in conjunction with the presence of another marker of clonal growth, such as somatic mutations within genes such as *ASXL1*, *EZH2*, *TET2*, *IDH1*, *IDH2*, *SRSF2*, or *SF3B1*, or the exclusion of reactionary (secondary) marrow fibrosis, is indicative of this diagnosis.

Criteria for minor PMF include the following:

- Increase in lactate dehydrogenase (LDH) activity.
- Anemia unrelated to comorbidities.
- Enlargement of the spleen on physical examination.
- Leukocytosis ≥ 11 × 10^9^/L.
- Leukoerythroblastosis.

To diagnose both prefibrotic and fibrotic phases of primary myelofibrosis, all of the major criteria and at least one minor criterion for a given type must be met. Secondary myelofibrosis may also develop on the basis of polycythemia vera (post-PV MF) or essential thrombocytosis (post-EV MF), especially in chronically ill patients. To establish this type of transformation, it is necessary to have a documented previous diagnosis of PV or ET according to WHO criteria, as well as a histological demonstration of marrow fibrosis grade 2–3 (on a 0–3 scale) or 3–4 (on a 0–4 scale). Additionally, at least two of the five supplementary criteria must be met: anemia below the age- and sex-specific reference range, decreased hemoglobin (>2 g/dL below the reference range), leukoerythroblastosis, progressive splenomegaly, increased LDH activity, and general symptoms manifested as fever, weight loss (>10% at six months), and recurrent night sweats [68,69,71].

Given the inherent challenges associated with bone marrow aspiration in the fibrotic phase of PMF (commonly referred to as an “empty biopsy”), a bone marrow trepanobiopsy is typically the primary diagnostic test. The histopathological examination of the trepanobioptate reveals a notable increase in the number of reticulin fibers, accompanied by mild to moderate collagen fibrosis. If the diagnosis is made in the early prefibrotic phase (approximately 30–40% of cases), in which the marrow remains rich in cellularity, the microscopic picture shows the presence of numerous neutrophils, as well as clusters of megakaryocytes with features indicative of quality disorders [2].

Furthermore, laboratory studies indicate an elevation in uric acid, bilirubin, and elevated alkaline phosphatase (ALP) and lactate dehydrogenase (LDH) activity. Moreover, cytometric analysis reveals an elevated proportion of hematopoietic stem cells (CD34+) in the peripheral blood. This parameter is not included in the WHO classification, but may prove useful in establishing the diagnosis, as evidenced by previous studies [68,70,71].

In the initial phase of the disease, designated the prefibrotic phase, thrombocytosis is observed, whereas in the advanced fibrotic phase, thrombocytopenia is evident. The number of leukocytes may vary, exhibiting a range from leukopenia to leukocytosis. Given the observed hyperthrombocytopenia, the clinical picture of PMF in the early stage may bear resemblance to that of spontaneous hyperthrombocytopenia. In order to ascertain prognosis, differential diagnosis of both disease entities is of significant importance at this stage. Additionally, primary myelofibrosis is characterized by the presence of anemia, with the microscopic evaluation of the blood smear revealing the presence of morphologically altered erythrocytes, shaped like a teardrop (lacrimocytes), and similar in size to erythrocytes of giant platelets. In order to differentiate between PMF and other hematologic malignancies, such as CML, PV, ET, MDS, CMML, lymphomas, or hairy cell leukemia, a comprehensive differential diagnosis is essential. In rare cases, solid tumors may also metastasize to the bone marrow. It is also important to consider the possibility of secondary marrow fibrosis resulting from other conditions, including mycobacterial infection, hyperparathyroidism, Paget’s disease, collagenosis, and chronic vitamin D deficiency [68,69,70,71,72,73].

The precise etiology of PMF remains uncertain. However, as with PV and ET, there is evidence of deregulation of the JAK/STAT signaling pathway. Genetic diagnosis, including cytogenetic and molecular testing, is important for diagnosis, treatment stratification, and prognosis. Because of the need to differentiate from CML, karyotype evaluation and FISH testing for *BCR*::*ABL1* translocations are essential. Karyotype abnormalities affect approximately 30–50% of diagnoses and most commonly include del (13q), del (20q), +8, +9, del (12p), and trisomy 1q. Molecular diagnosis includes mutational analysis of genes similar to ET and PV: *JAK2*, *CALR*, and *MPL*, especially the somatic mutation p.Val617Phe. Mutations within these genes are variously found in 40–50%, approximately 30%, and 5–10% of PMF patients. In so-called triple-negative cases (without mutations in the above-mentioned genes), which account for about 10–15% of PMF. Other pathogenic variants are sought within the remaining high molecular risk genes, such as *ASXL1*, *TET2*, *SRSF2*, *DNMT3A*, *U2AF1*, *EZH2*, *IDH1*, *IDH2*, *ET2*, *TP53*, *CBL*, *KRAS*, *NRAS, STAG2*, and *SRSF2* [73]. High-throughput NGS sequencing or targeted techniques using real-time quantitative PCR (real-time qPCR) or a more modern and sensitive variant of digital PCR (digital PCR) for canonical lesions are used [5,6,47,74].

The mutational status of the *CALR* gene—when occurring in conjunction with other mutations—has been demonstrated to possess considerable prognostic significance. Patients who lack a *CALR* gene mutation but possess an *ASXL1* gene mutation, designated *CALR*::*ASXL1*-positive, exhibit a markedly adverse prognosis and the shortest survival duration. This group may be eligible for bone marrow transplantation at an early stage of the disease. In contrast, patients with somatic *JAK2* mutations may be eligible for therapy based on *JAK* kinase inhibitors (e.g., ruxolitinib; fedratinib), which have demonstrated high therapeutic efficacy in advanced PMF in clinical trials. Of all myeloproliferative neoplasms, PMF is associated with the poorest prognosis and carries a high risk of blastic transformation to AML, which typically occurs in the advanced stages of the disease [70].

A study by Barbui et al. of 707 patients with PMF assessed the incidence and predictive factors for major cardiovascular disease. It was observed that in patients with PMF, the incidence was comparable to that reported for ET, and was also increased in elderly patients and those with the *JAK2* p.V617F mutation with associated leukocytosis. In addition, patients with PMF had a high rate of deep vein thrombosis affecting the legs, brain, and splanchnic area. In contrast to ET and PV, the incidence of myocardial infarction was lower relative to stroke [75].

The *JAK2* p.V617F mutation status was identified as an independent prognostic factor, which was further associated with leukocytosis. Both parameters identified groups of PMF patients with different risks of fatal and non-fatal cardiovascular complications [75].

## 7. Essential Thrombocythemia (ET)

Essential thrombocythemia vera (ET) is a subtype of MPNs that lacks the Philadelphia (Ph-) chromosome, along with polycythemia vera (PV) and primary myelofibrosis (PMF). The disease is defined by a pathological increase in the number of thrombocytes (PLT) in the peripheral blood above 450 G/l, which is not attributable to other conditions that can cause reactive thrombocytosis (termed secondary hyperplasia). Furthermore, the histological examination of the bone marrow reveals an excessive proliferation of megakaryocytes, accompanied by an increased number of their large and mature forms. Additionally, the blood smear demonstrates a higher percentage of giant platelets. In rare instances, spontaneous hyperplasia may evolve into acute myeloid leukemia or myelofibrosis (MF). The incidence of ET varies between 1 and 2.5 per 100,000 cases per year, depending on the population and age group under consideration. The incidence of this disease increases with age, with the median age of onset being approximately 60 years. However, cases have also been documented in individuals as young as 30 years of age. The disease is less prevalent among children and adolescents, with an incidence of approximately 0.09 per 100,000 cases per year. Epidemiological data indicate that women are twice as likely to be affected by this condition [68,69,70,71,72].

A classification system based on both large and small criteria is utilized for the diagnosis of ET in accordance with the most recent fifth edition of the World Health Organization’s 2022 guidelines. The large criteria are as follows:

- A platelet count ≥ 450 G/L.
- The presence of clonal proliferation of megakaryocytes, accompanied by an increased percentage of large, mature megakaryocytes with hyperlobulated nuclei, is a notable feature. There is no evidence of marked proliferation or a leftward shift in granulocytic and red cell lines. Infrequently, minimal reticulin fibrosis (≤ 1) is observed.
- Exclusion of other myeloproliferative neoplasms: PV, PMF, CML, CEL, and CNL.
- The presence of mutations in genes: mutations in the *JAK2*, *CALR*, or *MPL* genes.

Conversely, the minor criterion is the presence of an additional clonality marker, and in its absence, the exclusion of potential causes of secondary (reactive) hyperplasia. A diagnosis of spontaneous hyperplasia necessitates the presence of all major criteria, alternatively, the first three major criteria in conjunction with the minor criterion [74,75,76].

It is important to differentiate between spontaneous hyperplasia and other myeloproliferative neoplasms (CML, PV, and PMF) as well as myelodysplastic/myeloproliferative neoplasms (MDS/MPN-RS-T) that progress with an elevated platelet (PLT) count. It is of particular importance to be able to distinguish between ET and primary marrow fibrosis in the so-called pre-fibrotic phase, the clinical picture of which is very similar to that of ET. Spontaneous hyperplasia is often asymptomatic for an extended period, potentially leading to undetected cases over many years. A persistently elevated platelet count is most often identified fortuitously during routine complete blood count (CBC) examinations [68,70,71,73].

A peripheral blood smear reveals an elevated percentage of giant platelets, and upon physical examination, approximately 20% of patients present with an enlarged spleen (splenomegaly). Additionally, the diagnostic algorithm for ET encompasses the assessment of serum iron levels and acute-phase proteins, such as CRP. Additionally, molecular diagnostics is essential for the assessment of the mutational status of the three primary high-risk genes associated with the development of MPNs (*JAK2*, *CALR*, *MPL*). Extended molecular diagnostics comprises the analysis of over a dozen additional genes involved in MPN pathogenesis to varying degrees, employing next-generation sequencing (NGS). Additional high-risk molecular variants are then sought [47,69,77].

Of the three driver mutations listed, *JAK2* mutations are observed most frequently and affect about 50–60% of ET diagnoses. The second most common mutation (about 25% of diagnoses) is the *CALR* gene. Mutations of the *MPL* gene in ET patients are identified most rarely (about 3% of diagnoses) [43,44,45,46,47].

In addition, more than half of ET patients have alterations in other genes, including *ET2*, *ASXL1*, and *DNMT3A*. The occurrence of mutations in genes encoding specific splicing regulatory proteins (*SRSF2*, *SF3B1*, *U2AF1*, *ZRSR2*), chromatin structure modulators, epigenetic regulators, and proteins involved in cell signaling (e.g., *EZH2*, *IDH1*, *IDH2*, *CBL*, *KRAS*, *NRAS*, *STAG2*, *TP53*) is less prevalent. These are primarily observed in patients with advanced primary marrow fibrosis [5,17,43,44,45,46,74].

## 8. Chronic Neutrophilic Leukemia (CNL)

Chronic neutrophilic leukemia (CNL) is a rare MPN characterized by persistent neutrophilia, splenomegaly, and poor prognosis. It is diagnosed mostly in adult patients with a median age of 73 and a median survival rate of 2 years [78]. Studies on familial CNL showed that it may have a prolonged, asymptomatic period of granulocytosis [79]. Only a few cases in children have been reported and clinical criteria for diagnosis were the same as in adult CNL [80,81,82].

CNL is a clinically heterogeneous disease. It may present with incidental neutrophilic leukocytosis or chronic leukocytosis lasting up to 5 years. Common symptoms include fatigue, bone pain, easy bruising, gout, and pruritus. In clinical examination splenomegaly and hepatomegaly can be observed; however, lymph node involvement is uncommon. Many studies associate CNL with hemorrhagic diathesis and a higher incidence of cerebral hemorrhage connected with thrombocytopenia, platelet dysfunction, or infiltration of the vascular wall by neoplastic cells. Patients can show prolonged time of bleeding and platelet aggregation [78].

The diagnostic genetic signature for CLN is a mutation in the colony-stimulating factor 3 receptor, *CSF3R* located on chromosome 1 at position 1p34.3. The protein encoded by this gene is a receptor for cytokine, which is crucial in the process of differentiation and maturation of granulocyte precursors to neutrophils [83]. Mutation of *CSF3R* was detected in the majority (80%) of patients. However, the absence of this mutation does not exclude the diagnosis of CNL. The most common mutation in this gene is T618I, which causes ligand-independent activation of downstream kinase signaling pathways including the JAK/STAT pathway [84].

Studies show that up to 56% of CNL patients have additional *SETBP1* mutation. *SETBP1* is a gene located on chromosome 18 at position 18q12.3 and encodes a protein that binds to the SET nuclear oncogene. A mutation occurs in the SKI-homology region, causing protein overexpression, leading to the loss of tumor suppressor function and altered myeloid transcription factor expression. This mutation relates to a worse prognosis [85]. In most cases additional mutations, such as epigenetic modifiers (*TET2*, *ASXL1*) and spliceosome proteins (*SRSF2*, *U2AF1*) are identified; however, those are not exclusive for CNL and are observed in other MPNs [84].

Diagnostic criteria include peripheral blood granulocytes, at the segmented or band stage, constituting ≥80% of WBC. Leukocytosis, in cases without *CSF3R* mutation, is ≥25 × 10^9^/L, but the ICC suggests lowering the threshold for leukocytosis in cases with a mutation in this gene to ≥13 × 10^9^/L. Moreover, neutrophil precursors should represent less than 10% of WBC. The same criteria apply to monocytes. Circulating blasts in peripheral blood are rarely observed [6,86]. Hypercellularity of the bone marrow is shown due to increased neutrophilic granulopoiesis. There are less than 5% of blasts in bone marrow [5,39]. CNL can be diagnosed in cases that do not meet the diagnostic criteria for other MDS such as *BCR*::*ABL1*-positive CML, PV, and ET [6].

Distinguishing CNL from atypical CML or other MDS may be challenging. According to the WHO and ICC, the diagnostic criteria for differentiating CNL are peripheral blood white blood cell count and no dysgranulopoiesis (the WHO suggests ≥25 × 10^9^/L, but ICC ≥13 × 10^9^/L., normal neutrophil granulocytes maturation with hypercellular bone marrow, the presence of the *CSF3R* T618I mutation or another activating *CSF3R* mutation, and the absence of the Philadelphia chromosome [5,6].

Henrik Hasle et.al reported the case of a 15-year-old girl presenting with epistaxis, easy bruising, and fatigue. Examination showed splenomegaly. Blood tests showed severe thrombocytopenia and leukocytosis with mature neutrophils. PCR for *BCR*::*ABL1* was negative. A bone marrow transplant was performed, and after 6 years, the patient had a normal hematological constitution [80]. Lawrence J. Druhan et.al reported the case of a girl with leukocytosis at birth, at the age of 4, an ultrasound showed splenomegaly. At 10 years of age, splenomegaly increased. Cytogenetics and FISH were normal. PCR for *BCR*::*ABL1* was negative. Sanger sequencing showed a *CSF3R* T618I mutation. The *ASXL1*, *SETBP1*, and *TET2* genes were valid [81]. Vedat Uygun et.al reported a patient that was referred to the hematology department for recurrent pulmonary infections and accompanying leukopenia at the age of 8. The patient was diagnosed with Fanconi anemia (FA). At the age of 12, thrombocytopenia was resolved, and the WBC count increased to 15 × 10^9^/L, but in two months, it increased to 63.8 × 10^9^/L. Tests reported genetic markers t (9,22), *JAK2, PDGFR*, *FGFR1*, *PCM1*::*JAK2*, and t (8,21) as negative, but mutation *CSF3R* T618I was detected. Confirmation of the mutation, splenomegaly, and normal granulocyte maturation led to the CNL diagnosis. To avoid progression to AML, HSCT was performed [82].

## 9. Chronic Eosinophilic Leukemia (CEL)

Chronic eosinophilic leukemia (CEL) is a rare, clonal eosinophilic disorder that affects both adults and children, though pediatric cases are notably uncommon. The eosinophils infiltrate and damage organs, posing significant health risks. The disease’s pathophysiology often involves genetic mutations or chromosomal abnormalities that activate tyrosine kinase signaling pathways, leading to excessive eosinophil growth. Advances in molecular diagnostics and targeted therapies have allowed for improved classification and treatment of CEL, particularly with the use of tyrosine kinase inhibitors (TKIs) such as imatinib [87,88,89,90,91].

CEL in pediatric patients can be particularly aggressive, with a significant risk for organ damage due to eosinophilic infiltration. Molecular analysis has revealed several key mutations associated with CEL, including gene fusions involving *PDGFRB*, *PDGFRA*, and *FGFR1*, as well as rare occurrences of *JAK2* mutations, such as *JAK2* V617F. These genetic changes lead to constitutive activation of tyrosine kinase pathways, driving eosinophil proliferation and survival. In pediatric cases, chromosomal translocations, such as t (1;5) (q21; q33), can result in rare fusions like *TPM3*::*PDGFRB* [87,89,92].

The identification of these genetic fusions has had significant therapeutic implications, allowing for the application of targeted tyrosine kinase inhibitors like imatinib. Imatinib specifically inhibits *PDGFRB*-related fusions, making it particularly effective in cases where these mutations are present [89].

CEL diagnosis relies on evidence of persistent hypereosinophilia (HE), defined as an eosinophil count above 1.5 × 10^9^/L, and molecular or cytogenetic markers confirming clonality [93].

Table 5 shows the key diagnostic steps in CEL.

A study by Li et al. reported on an 8-year-old boy with CEL harboring a *TPM3*::*PDGFRB* fusion, resulting from a t (1;5) (q21; q33) chromosomal translocation. The presence of this fusion was confirmed through sequencing, and an RT-qPCR assay was developed to monitor the fusion transcript levels. This real-time monitoring provided a valuable tool for assessing treatment efficacy, as imatinib rapidly reduced the patient’s leukemic burden and *TPM3*::*PDGFRB* transcript levels. The case emphasizes the importance of molecular diagnostics in managing pediatric CEL, offering a pathway to tailored, effective treatments and better prognosis [87,92,93]

For CEL cases involving *PDGFRB* fusions, imatinib is the standard of care. This TKI specifically inhibits *PDGFRB*-driven signaling, providing targeted control of eosinophil proliferation and improving patient outcomes. In pediatric patients, the dosage of imatinib may be adjusted to balance efficacy and minimize side effects. In cases lacking actionable mutations, corticosteroids are employed to control eosinophil-mediated inflammation. Hydroxyurea is an alternative for patients who are steroid-refractory or require long-term control. The use of RT-qPCR for ongoing monitoring of *PDGFRB* fusion transcripts, as demonstrated in the *TPM3*::*PDGFRB* case, enables clinicians to assess treatment efficacy in real time, facilitating timely adjustments to therapy [91,93].

The study by Legrand et al. (2013) provides valuable insights into the management and outcomes of *FIP1L1*::*PDGFRA*-associated chronic eosinophilic leukemia (F/P+ CEL), especially regarding the efficacy and dosing strategies of imatinib, a targeted therapy that has revolutionized treatment for this condition [94]. The *FIP1L1*::*PDGFRA* fusion gene, resulting from a specific chromosomal deletion, creates a constitutively active tyrosine kinase. This overactivity drives eosinophil proliferation [94].

In the cohort of Fijolek’s research, 57% of patients had skin involvement, 52% had splenomegaly, 45% had lung involvement, and 34% had cardiac disease. Pediatric CEL patients might present with similar organ involvement due to eosinophil infiltration, which causes tissue damage and could be seen in the gastrointestinal tract, lungs, or heart. Elevated levels of vitamin B12 and tryptase were common in F/P+ CEL and may also aid in diagnosing pediatric CEL cases, providing indirect markers of eosinophil activation and infiltration severity [95].

Advances in molecular understanding have led to the development of novel therapeutics targeting pathways involved in eosinophil survival and proliferation. In CEL and other eosinophilic disorders, the IL-5 pathway and the JAK-STAT signaling cascade have become critical therapeutic targets:

- *JAK* inhibitors: in cases where *JAK2* mutations are present, *JAK* inhibitors, such as ruxolitinib, are being explored. Ruxolitinib’s inhibition of the JAK2-STAT pathway reduces eosinophil survival signals mediated by IL-5 and GM-CSF, potentially benefiting patients with refractory or advanced CEL.
- IL-5 pathway inhibitors: agents such as benralizumab and depemokimab, which target IL-5 or its receptor, are in clinical trials. These agents can suppress eosinophil proliferation and have shown promise in reducing the need for corticosteroids and mitigating eosinophil-associated tissue damage [88,93].

For cases unresponsive to TKIs or where genetic testing does not reveal actionable mutations, more intensive treatments, including cytotoxic chemotherapy or hematopoietic stem cell transplantation (HSCT), may be considered. These approaches are reserved for aggressive, refractory cases and carry significant risk, particularly in pediatric patients. However, HSCT offers a potential cure in selected patients [88,93].

## 10. Juvenile Myelomonocytic Leukemia (JMML)

Juvenile myelomonocytic leukemia (JMML) is a Ph-negative, rare myelodysplastic/myeloproliferative neoplasm overlap syndrome that affects young children, characterized by abnormal overproduction of myeloid progenitor cells and monocytes that does not exceed 20% in the bone marrow and peripheral blood. This condition progresses aggressively and often has a poor prognosis. Cell differentiation in JMML shifts predominantly towards monocytic lineages, with progenitor colonies showing a range of stages, including blasts, pro-monocytes, monocytes, and macrophages [93]. Additionally, JMML progenitor cells show hypersensitivity to granulocyte-macrophage colony-stimulating factor (GM-CSF), due to dysregulated activation of the Ras signaling pathway. Overproduction within the myeloid lineage suppresses other cell lines, often causing anemia and thrombocytopenia in affected patients [96].

JMML represents approximately 1–2% of all pediatric leukemias, with an incidence of about 1.2 cases per million people per year. The median age at diagnosis is two years, with a noticeable male predominance (male-to-female ratio of 2.5). Around three-fourths of cases are diagnosed before the age of three and 95% are identified by age six [96].

The pathogenetic mechanism of JMML is the deregulation of the intracellular Ras signal transduction pathway, occurring in at least 90% of cases and caused by mutations in one of five genes: *PTPN11*, *NRAS*, *KRAS*, *NF1*, or *CBL* [97].

The most common mutation in the Ras pathway associated with JMML is the somatic *PTPN11* mutation in exons 3 or 13, present in up to 35% of JMML patients. The *PTPN11* gene encodes the Src-homology tyrosine phosphatase 2 (SHP2), which consists of two Src-homology 2 (N-SH2 and C-SH2) domains and a catalytic phosphatase domain. Germline mutations in Noonan syndrome (NS), as well as somatic mutations in JMML, affect residues within the N-SH2 or phosphatase domains [98]. Mutations associated with JMML are more aggressive, as they involve stronger gain-of-function alleles, leading to increased phosphatase activity and a more severe disease course. In contrast, germline mutations in NS tend to be less severe, which may account for cases where myeloproliferative symptoms resolve spontaneously [96].

*NRAS* or *KRAS* point mutations are present in about 25% of JMML patients. JMML with somatic *RAS* mutations generally has an aggressive course, though a small subset of patients with somatic *NRAS* mutations may experience spontaneous remission. *RAS* proteins function as signaling molecules, cycling between an active GTP-bound state (RAS-GTP) and an inactive GDP-bound state (RAS-GDP). This switch is regulated by guanine nucleotide exchange factors, which convert RAS-GDP to active RAS-GTP, and by the hydrolysis of RAS-GTP back to RAS-GDP through an intrinsic GTPase activity in *RAS*, which is enhanced by GTPase-activating proteins (GAP) like NF-1. RAS-GTP promotes downstream cellular responses, including cell proliferation, differentiation, and survival. Mutations in *RAS* result in impaired GTPase activity and reduced responsiveness to GAPs, causing an accumulation of active RAS-GTP [96,99].

A germline *KRAS* mutation found in Noonan syndrome (NS) can lead to a mild JMML-like myeloproliferative disorder. These NS-associated mutations are distinct from those seen in cancers and leukemias; for instance, the *KRAS* T58I allele (found in NS) has milder effects compared to the classic oncogenic *KRAS* G12D mutation or wild-type *KRAS*. Additionally, *RAS* mutations can also lead to a lymphoproliferative condition known as *RAS*-associated lymphoproliferative disorder (RALD), which shares some clinical features with JMML [96].

The *NF-1* gene functions as a tumor suppressor, producing the protein neurofibromin, a GTPase-activating protein that regulates *RAS* activity. A clinical diagnosis of *NF-1* can be established in up to 11% of JMML patients, while approximately 15% have identifiable mutations in the *NF-1* gene. Children with both *NF-1* and JMML are often older and present with a more aggressive disease course. These patients carry one defective *NF-1* allele in their germline, and JMML cells in these patients exhibit homozygous *NF-1* inactivation due to somatic loss of the remaining normal allele in leukemic cells, leading to *RAS* hyperactivation. Most *NF-1* mutations are autosomal dominant missense or nonsense mutations, but other types, including small insertions or deletions, as well as large deletions or chromosomal translocations involving *NF-1*, have also been identified [96].

The *CBL* gene plays a role in regulating the *RAS* pathway by downregulating the Janus kinase (*JAK2*) pathway. The *CBL* mutations, present in 10–15% of JMML patients, are typically missense germline mutations [100]. When somatic loss of heterozygosity occurs, it grants malignant potential to these mutated cells. In JMML cases with *CBL* mutations, the only recurring genetic change is copy-neutral isodisomy at 11q23.3, where the *CBL* gene is located, without other accompanying mutations. The prognosis in *CBL*-mutated JMML can vary, with some cases spontaneously resolving while others progress aggressively. Homozygous *CBL* mutations arising as germline events often correspond to a JMML variant prone to spontaneous remission. These homozygous germline *CBL* mutations may also lead to *CBL* syndrome, a condition marked by neurological symptoms, vasculitis, and features like Noonan syndrome [101,102].

Fewer than 10% of JMML cases lack the five canonical driver mutations. Other fusion mutations observed in children with myeloproliferative disorders with the use of next-generation sequencing technologies include *ALK*, *ROS1*, *FIP1L1*::*RARA*, *HCMOGT1*::*PDGFRB*, *NDEL1*::*PDGFRB*, and *NUP98*::*HOXA11*. In the International Consensus Classification, cases in nonsyndromic patients that phenotypically resemble JMML but do not have a Ras pathway mutation are classified as JMML-like [6].

Splenomegaly is observed in nearly all cases of JMML, hepatomegaly is also common. Skin rash and lymphadenopathy, resulting from leukemic infiltration, are observed in approximately 50% and 80% of JMML cases, respectively. Respiratory symptoms such as dry cough, tachypnea, and interstitial infiltrates seen on chest radiographs indicate peribronchial and interstitial pulmonary infiltrates. Gastrointestinal involvement can lead to complications such as bloody diarrhea and increased susceptibility to gastrointestinal infections. Central nervous system involvement in JMML is very rare [97].

A peripheral blood (PB) smear typically reveals leukocytosis, monocytosis, and the presence of immature monocytes, along with myelocytes, metamyelocytes, and nucleated red blood cells. Occasionally, a few blasts may be observed. Thrombocytopenia and anemia are frequently present. Bone marrow (BM) findings in JMML, while not diagnostic, support the diagnosis [97]. The blast count in the marrow can be moderately elevated but does not reach the levels associated with acute leukemia. In about two-thirds of cases, megakaryocytes are reduced in number or absent. A notable characteristic of many JMML cases is the increased synthesis of hemoglobin F (HbF) [6]. Autoimmunity signs, such as the presence of antinuclear antibodies or a positive anti-globin test, can be detected in up to 25% of children with JMML, while hypergammaglobulinemia is present in more than half of the affected individuals. A combination of young age, splenomegaly, skin lesions, the presence of myeloid and erythroid precursors in the peripheral blood, and/or elevated HbF levels should alert the pediatric oncologist to consider JMML and proceed with targeted diagnostic tests [8].

The diagnostic criteria for JMML based on the 5th Edition of the WHO Classification of Hematolymphoid Tumors guidelines are presented in Table 6 [5].

## 11. MPN-NOS/MPN-U

According to the WHO 2022, the last of the disease entities classified under the group of “myeloproliferative neoplasms” is MPN, not otherwise specified (MPN-NOS). In contrast, the latest ICC classification distinguishes this entity under the name—MPN, unclassifiable (MPN-U). Regardless of the name, clinically they mean the same thing, i.e., the absence of an identifiable biological marker, a genetic marker, which would classify a diagnosed MPN into any of the subtypes already known. A myeloproliferative neoplasm that does not meet the WHO or ICC criteria for other MPNs is a very rare MPN, poorly described and defined. It poses a challenge to clinicians, due to the lack of guidelines for therapy and patient monitoring, because of the absence of prognostic markers [103,104].

## 12. Conclusions and Future Directions

Early diagnosis of MPN in children and young adults still poses a challenge to the physician, so detailed genetic screening may provide a more accurate understanding of the individual disease as well as on population level. Despite the fact that MPNs are currently diagnosed with greater frequency than in the past, it still remains a very rare disease. The majority of experience in the treatment of these conditions comes from the treatment of adult patients.

To obtain a better understanding of the disease in children, it is essential to conduct further studies on larger patient groups with longer follow-up periods. This will facilitate a more comprehensive understanding of the symptom burden, the actual incidence of hemostatic complications, disease transformation, and the impact on the life quality in young patients. In contrast to adult patients, it is necessary to consider other factors, such as the long-term impact of abnormal blood counts on the vascular system, specific indicators of cardiovascular risk factors in the general population, fertility and pregnancy complications, the elevated risk of cancer, and the potential for long-term use of cytoreductive drugs. These considerations should prompt physicians to develop a tailored therapeutic approach to MPN in children. It is also of great importance to establish an early and precise diagnosis of MPN in children, as this will facilitate the collection of data on long-term outcomes and enable the identification of the safest therapies. It is imperative to define appropriate diagnostic standards and establish appropriate treatment parameters for these young patients. This will enable healthcare professionals to counsel and guide families and young adults in making informed decisions about their care. The aim of this study is to summarize the knowledge about myeloproliferative neoplasms to help develop future standards and guidelines for the diagnosis and treatment of MPN in children and adolescents.

## Figures and Tables

**Table 1:** Classification WHO of MPN [5].

WHO 2022 Myeloproliferative Neoplasms
Chronic myeloid leukemia
Polycythemia vera
Essential thrombocythemia
Primary myelofibrosis
Chronic neutrophilic leukemia
Chronic eosinophilic leukemia
Juvenile myelomonocytic leukemia
Myeloproliferative neoplasm, not otherwise specified

**Table 2:** Classification ICC of MPN [6].

International Consensus Classification of Myeloid Neoplasms 2022
Chronic myeloid leukemia
Polycythemia vera
Essential thrombocythemia
Primary myelofibrosis
Early/prefibrotic primary myelofibrosis
Overt primary myelofibrosis
Chronic neutrophilic leukemia
Chronic eosinophilic leukemia, not otherwise specified
Pediatric and/or germline mutation-associated disorders
Juvenile myelomonocytic leukemia
Juvenile myelomonocytic leukemia-like neoplasms
Noonan syndrome-associated myeloproliferative disorder
Refractory cytopenia of childhood

**Table 3:** Biological markers of MPN.

Gene	Locus	Exon Count	Type of Mutations	Protein	Protein Function	Consequences in MPN	Reference
Signaling MPN driver							
*JAK2*	9p24.1	28	*JAK2* V617F (exon 14), *JAK2* exon 12	Janus Kinase 2 (tyrosine kinase)	tyrosine kinase, forming JAK-STAT signaling pathway	increased bone marrow hematopoietic cell production	[8,9,10,11]
*MPL*	1p34.2	12	point mutations: *MPL* W515L, *MPL* W515K, *MPL* S505N	thrombopoietin receptor	regulates the production of multipotent hematopoietic progenitor cells and platelets	proliferation of the myeloid lineage in bone marrow, mainly platelet production	[2,9,12,13,14]
*CALR*	19p13.13	9	deletion in exon 9, insertion in exon 9	calreticulin	glycoprotein folding, calcium cation storage in the endoplasmic reticulum	increased number of platelets	[2,9,15,16,17,18,19]
DNA methylation							
*TET2*	4q24	12	somatic missense mutations, insertion-deletion mutations, somatic nonsense mutations; usually exon 3 or 11	enzyme ten-eleven- translocatin-2	epigenetic gene regulation of DNA-demethylation	extreme hypotheses: induction of the occurrence of *JAK2* mutation (in ET) or no significance	[20,21,22,23,24,25]
*DNMT3A*	2p23.3	26	*DNMT3A* R882C	DNA methyltransferase 3A	epigenetic gene regulation DNA-methylation	often appears during treatment; unclear hypotheses	[22,26,27,28,29,30]
*IDH1*	2q33.3	12	point mutations: *IDH1* R132S, *IDH1* R132C, *IDH1* R132G	isocitrate dehydrogenase 1	oxidative decarboxylation of isocitrate to α-ketoglutarate; *IDH1* (cytoplasm and peroxisome) *IDH2* (mitochondria)	disease progression and leukemic transformation, inhibiting the demethylating activity of *TET2*	[22,31,32,33,34]
*IDH2*	15q26.1	12	point mutations: *IDH2* R140Q, *IDH2* R140W, *IDH2* R172G	isocitrate dehydrogenase 2			
Maintenance of chromatin structure							
*ASXL1*	20q11.21	13	mutation in exon 12	additional sex combs-like 1	methylation of histone H3K27, regulation of expression of *HOX* genes, remodeling of nucleosome	transformation of myeloid cells by inhibiting methylation	[32,35,36,37,38]
*EZH2*	7q36.1	20	loss-of-function mutations	enhancer of zeste 2 repressive complex 2 subunit	methylation of histone H3K27	chromatin relaxation and transcriptional availability in aberrant hematopoiesis	[32,39]
Splicing							
*SF3B1*	2q33.1	27	point mutations: *SF3B1* K700E, *SF3B1* H662Q	RNA-splicing factor 3B subunit 1	spliceosome assembly	phenotypic change	[22,40]
*SRSF2*	17q25.1	5	point mutations: *SRSF2* P95H, *SRSF2* P95L	serine/arginine-rich pre-RNA splicing factor 2		promotion of leukemogenesis, myelodysplasia with proliferative features, identification of a frequent overlap of mutations in *IDH2*	[20,32,41]
*U2AF1*	21q22.3	11	point mutations: *U2AF1* S34F, *U2AF1* S34Y	U2 small nuclear auxiliary factor 1		defect in splicing, significantly shorter overall survival	[32,42]

**Table 4:** The tests necessary for the diagnosis of PV [62,63,64,65].

Type of Study	Substantiation
Determination of *JAK2* V617F gain-of-function mutation carrier status.	Marker of polycythemia vera, used to distinguish PV from post-PV but not from ET and PMF.
In the absence of mutations *JAK2* V617F; determination of *JAK2* exon 12 or *TET2* mutation carrier status, *ASXL1, SRSF2*. Mutations that worsen the prognosis include *IDH2*, *RUNX1* and *U2AF1*.	Determining the presence of mutations in the *JAK2* gene allows for distinguishing PV from secondary forms of polycythemia. Additionally, determining the mutation status in the *CARL* and *MPL* genes to distinguish it from other myeloproliferative diseases.
Erythropoietin level in serum.	Reduced serum EPO concentration distinguishes PV from secondary polycythemia.
Blood count: ↑ RBC, ↑ HCT, ↑ HGB, ↑ FAG Also possible ↑ WBC and/or ↑PLT	Blood count is the basic test necessary to diagnose PV.
Abdominal ultrasound showing splenomegaly.	Disease progression rate.
Bone marrow biopsy: panmyelosis (erythroid, granulocytic, and/or thrombocytic hyperplasia).	Bone marrow biopsy does not need to be performed in patients with a confirmed *JAK2* V617F gene mutation.
Cytogenetic tests: FISH: Exclusion of *BCR*::*ABL* fusion t(9;22)(q34;q11) Patient karyotype assessment: Most common karyotype abnormalities: trisomy 8, trisomy 9, trisomy 1q, trisomy 9p deletion: 12p, Y, 20q monosomy 7q31	Additional diagnostics are helpful in differentiating PV from ET and PMF and provide information about the patient’s prognosis and possible classification for more stringent treatment.
FAG—granulocyte alkaline phosphatase, ↑ increased	

**Table 5:** Key diagnostic in CEL [93].

Peripheral Blood and Bone Marrow Analysis	Morphologic Evaluation Aids in Identifying Dysplastic or Immature Eosinophils Characteristic of CEL.
Genetic and molecular testing	Detects chromosomal translocations and mutations, particularly, *PDGFRB*, *PDGFRA*, and *FGFR1* fusions, which guide therapeutic decisions. The presence of *JAK2* mutations, although rare, highlights the role of the JAK-STAT pathway and provides an additional treatment target.
Real-Time PCR (RT-qPCR)	In the case of *TPM3*::*PDGFRB* fusion, RT-qPCR allows for continuous monitoring of fusion transcripts, providing a real-time measure of disease response to treatment.
Flow cytometry and immunophenotyping	Differentiates CEL from other hematologic neoplasms, including reactive eosinophilia and acute leukemia.
Organ-specific imaging and functional assessment	Given the risk of organ damage, imaging (e.g., echocardiography) and functional assessments are essential in evaluating the extent of disease progression.

**Table 6:** Diagnostic criteria WHO of JMML [5].

Clinical, Hematologic, and Laboratory Criteria (All Criteria are Required for Diagnosis)
1. Peripheral blood monocyte count is ≥ 1 × 10^9^/L
2. Blasts and promonocytes constitute < 20% of peripheral blood and bone marrow
3. Clinical evidence of organ infiltration, most commonly splenomegaly
4. Absence of the *BCR*::*ABL1* fusion gene
5. Absence of a *KMT2A* rearrangement
Genetic criteria (1 criterion is sufficient for diagnosis)
1. A variant in a component or a regulator of the canonical *Ras* pathway: a. A clonal somatic variant in *PTPN11*, *KRAS*, or *NRAS* (a) b. A clonal somatic or germline variant in *NF1* and a loss of heterozygosity or compound heterozygosity in *NF1* c. A clonal somatic or germline variant in *CBL* and a loss of heterozygosity in *CBL* (b)
2. A noncanonical clonal *RAS* pathway pathogenic variant (c) or fusions that activate genes located upstream of the *RAS* pathway, such as *ALK*, *PDGFRB*, and *ROS1*
Other criteria (2 or more are required for diagnosis)
1. Circulating myeloid (promyelocytes, myelocytes, metamyelocytes) and erythroid precursors
2. Increased hemoglobin F for age
3. Thrombocytopenia with hypercellular bone marrow, often with megakaryocytic hypoplasia. Dysplastic features may or may not be evident
4. Myeloid progenitors the hypersensitive to GM-CSF (detected by clonogenic assays or by measuring STAT5 phosphorylation in the absence or with a low dose of exogenous GM-CSF)

(a) Germline variants in *PTPN11*, *KRAS*, or *NRAS* (which cause Noonan syndrome) may lead to JMML-like transient myeloproliferative disorder. (b) Occasional cases have heterozygous splice-site variants, (c) Such as *RRAS* or *RRAS2*.
